# The cushion–star *Parvulastra exigua* in South Africa: one species or more?

**DOI:** 10.3897/zookeys.524.6145

**Published:** 2015-09-30

**Authors:** Robyn P. Payne, Charles L. Griffiths, Sophie von der Heyden, Erich Koch

**Affiliations:** 1 Department of Biological Sciences, University of Cape Town, Private Bag X3, Rondebosch 7701, South Africa; 2 Marine Research Institute, University of Cape Town, Private Bag X3, Rondebosch 7701, Cape Town, South Africa; 3 Evolutionary Genomics Group, Department of Botany and Zoology, Stellenbosch University, Private Bag X1, Matieland, 7602, South Africa; 4 Present address: Department of Biodiversity and Conservation Biology, University of the Western Cape, Private Bag X17, Bellville 7535, South Africa

**Keywords:** Cryptic species, gonopore, *Parvulastra dyscrita*, *Parvulastra exigua*, *Patiriella*, starfish

## Abstract

The cushion–star *Parvulastra exigua* (Lamarck, 1816) is a widely distributed member of the temperate intertidal fauna in the southern hemisphere. In South Africa, it occurs in sympatry with the endemic *Parvulastra dyscrita* (Clark, 1923), the two species being differentiated predominantly by gonopore placement. Several recent studies have suggested that there may be additional cryptic species within the *Parvulastra exigua* complex in South Africa, based variously on color morphology, genetic evidence and the differential placement of the gonopores. This paper attempts to resolve whether one or more species are represented within *Parvulastra exigua*. A total of 346 *Parvulastra exigua* and 8 *Parvulastra dyscrita* were collected from sites on the west and south–west coasts of South Africa; morphological, anatomical and genetic analyses were performed to determine whether cryptic species and/or *Parvulastra exigua* specimens with aboral gonopores were present. Results show that neither cryptic species nor *Parvulastra exigua* specimens with aboral gonopores occur at these sites. This study thus refutes previous claims of the existence of aboral gonopores in South African *Parvulastra exigua*, and suggests that a single species is represented. The distinction between *Parvulastra exigua* and *Parvulastra dyscrita* is also confirmed, and features separating these two species are clarified and documented.

## Introduction

The dwarf cushion–star *Parvulastra exigua* (Lamarck, 1816) is a prominent and widespread member of the temperate intertidal fauna in the southern hemisphere (Hart et al. 2006), occurring along the entire southern coastline of Africa from Namibia to Mozambique, in southeastern Australia and on several oceanic islands (Clark and Downey 1992). In South Africa, *Parvulastra exigua* occurs in sympatry with another endemic cushion–star, *Parvulastra dyscrita* (Clark, 1923), which has a larger adult size, occurs in lower densities and is found predominantly subtidally along the south and east coast, between False Bay and East London (Branch et al. 2010). *Parvulastra dyscrita* has a complex and intertwined taxonomic history with *Parvulastra exigua* (Table 1), which in part was driven by their morphological and ecological similarities. However, *Parvulastra exigua* and *Parvulastra dyscrita* have now been confirmed as two separate species in an unpublished thesis by Dunbar (2006), based on molecular and morphological (external gonopore position) evidence.

**Table 1.** The taxonomic history of *Parvulastra exigua* and *Parvulastra dyscrita*.

Step in taxonomic history	Performed by
*Parvulastra exigua* first described as *Asterias exigua*.	Lamarck (1816)
*Asterina exigua* Lamarck found to be conspecific with *Asterina kraussii* Gray and *Asteriscus pentagonus* Müller & Troschel.	Perrier (1875)
Oral gonopore placement of *Asterina exigua* first noted. Oral gonopore placement of *Asterina exigua* confirmed.	Whitelegge (1889) Mortensen (1921)
*Asterina exigua* moved into the new genus *Patiriella* (often ignored by later authors).	Verrill (1913)
A new species with aboral gonopores, *Asterina dyscrita* described; suggestion made that it may only be a variety of *Asterina exigua*.	Clark (1923)
*Asterina dyscrita* placed into synonymy with *Asterina exigua*.	Mortensen (1933)
Asterina (Patiriella) exigua reviewed; it was proposed that there was a second species within *exigua* with aboral gonopores.	Dartnall (1971)
It was suggested that the species with aboral gonopores was *Asterina dyscrita* and moved to the genus *Patiriella* due to morphological similarity with *Patiriella exigua*.	Clark (1974)
*Patiriella exigua* and *Patiriella dyscrita* moved to the new genus *Parvulastra* which is distinguished from *Patiriella* based on ray width, ray plate alignment and is supported by previous molecular studies conducted by Waters et al. (2004).	O’ Loughlin and Waters (2004)
*Parvulastra exigua* and *Parvulastra dyscrita* were confirmed to be two separate species based on morphological (external gonopore position) and molecular evidence (mtDNA COI). Another species that only occurs in Kommetjie was recognized within *Parvulastra exigua*, but no species description was recorded.	Dunbar (2006)

**Figure 1.**
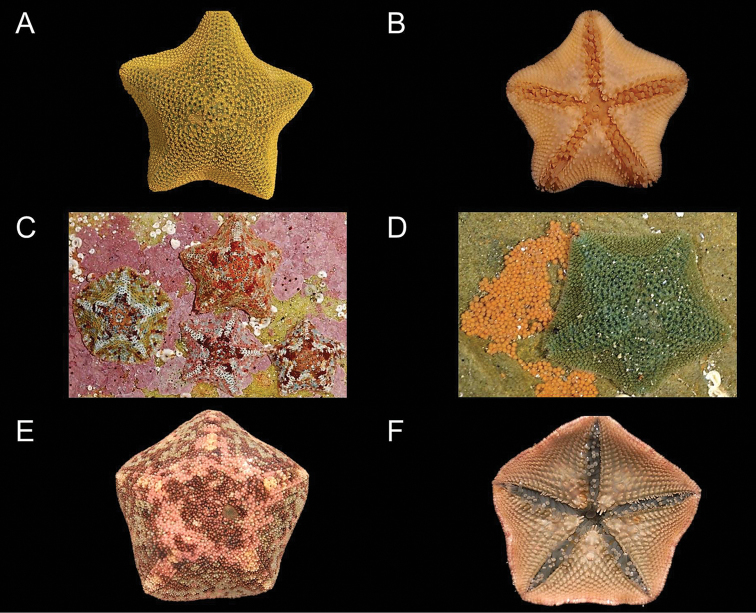
Abactinal (**A**) and actinal (**B**) view of *Parvulastra exigua*, with equivalent views of *Parvulastra dyscrita* (**E, F**), with the *Parvulastra exigua* mottled color morph found on the east coast of South Africa (**C**) and an adult *Parvulastra exigua* laying sticky eggs via oral gonopores onto the underside of a rock (**D**). All photos by C.L. Griffiths; individuals not to scale, with approximate sizes given in Table 5.

While the larger *Parvulastra dyscrita* has a fairly consistent mottled coloration (Fig. 1), *Parvulastra exigua* demonstrates a high degree of color variation, with two major color morphs in South Africa demonstrating an allopatric distribution (Fig. 1; Branch et al. 2010). The distribution patterns of these color morphs were studied in detail by Dunbar (2006), who noted a strong spatial divergence separated by a narrow zone of color morph sympatry around Cape Point. On the west coast, *Parvulastra exigua* were of a uniform khaki green color (similar to Australian populations), while more brightly colored, variegated individuals dominated along the east and south coasts. Dunbar (2006) also found the color morphs to demonstrate some degree of ecological divergence. The mottled morph is found predominantly in the high tidal zone within protected, bare rock, algae–encrusted and under boulder habitats with few algal tufts, while the green and intermediate color morphs were found in the mid tidal zone, with the green color morph inhabiting under boulder and bare rock habitats and tidal pools with little/no algae canopy and coralline algae. Similarly, the intermediate color morph also predominantly inhabited bare rock tidal pools, with little coralline algae, but also avoided those with algal tufts and an algal canopy.

Contrary to her expectations, Dunbar (2006) found no evidence of genetic separation between the two major color morphs, with perhaps temperature and/or predation maintaining the observed color polymorphism. In addition, a highly divergent haplotype was identified in twelve Kommetjie specimens, indicating the presence of a reproductively isolated cryptic species within a very narrow geographic range (Dunbar 2006). These individuals were documented as members of the intermediate color morph and exhibited a unique reddish–orange coloration (Dunbar 2006). Apart from this relatively subjective difference in color morph, Dunbar (2006) noted that this cryptic species appears morphologically similar to *Parvulastra exigua*, especially with regards to the presence of oral gonopores, but was found to be more closely related to the outgroup taxa *Parvulastra parvivipara* (Keough and Dartnall 1978) and *Parvulastra vivipara* (Dartnall 1969). Dunbar (2006) went on to suggest that this Kommetjie lineage should be classed as a new species, but to the authors’ knowledge, no such species description was ever prepared.

The major differentiating features between *Parvulastra exigua* and *Parvulastra dyscrita* are the position of the gonopore and reproductive mode. *Parvulastra exigua* is an ovipositor that spawns predominantly from August to October (Lawson–Kerr and Anderson 1978; Byrne 1992). The sticky egg masses are laid via oral gonopores (Lawson–Kerr and Anderson 1978) on the undersides of boulders and give rise to distinct lecithotrophic benthic larvae (Byrne and Anderson 1994). By contrast *Parvulastra dyscrita* releases eggs into the water column via aboral gonopores, where they hatch into planktonic larvae.

However, in 2006, Hart et al. externally examined various preserved specimens of *Parvulastra exigua* from museum collections from South Africa, southern Australia and several islands (St. Helena, Amsterdam, St. Paul and Kerguelen) for evidence that some *Parvulastra exigua* populations might include cryptic species with a different mode of reproduction. Overall, 33% (21% in South Africa) of the *Parvulastra exigua* specimens examined (excluding individuals in which gonopore position was uncertain) were reported to have aboral gonopores, with such individuals occurring predominantly in South Africa and the St. Paul, Amsterdam and St. Helena islands. Hart et al. (2006) also analyzed mitochondrial DNA sequence data from the study by Waters and Roy (2004), leading them to tentatively suggest that a cryptic species of *Parvulastra exigua* (or more) exist in South Africa, probably with aboral gonopores. Moreover, Dartnall and Byrne (unpublished observation cited in Colgan et al. 2005) proposed the presence of a cryptic species with aboral gonopores in South Africa and a few oceanic islands.

The studies of both Dunbar (2006) and Hart et al. (2006) point to the possibility (and presence) of cryptic species within *Parvulastra exigua*, yet with the absence of specimens from both studies and the lack of resolution on gonad structure, this remains unresolved. Therefore, the aim of this study was to resample *Parvulastra exigua* populations on either side of the morphological overlap to confirm whether genetically distinct specimens and/or *Parvulastra exigua* specimens with aboral gonopores are in fact present. To do this, mtDNA COI gene as well as geometric morphometric approaches were utilised.

## Methods

### Specimen collection

**Figure 2.**
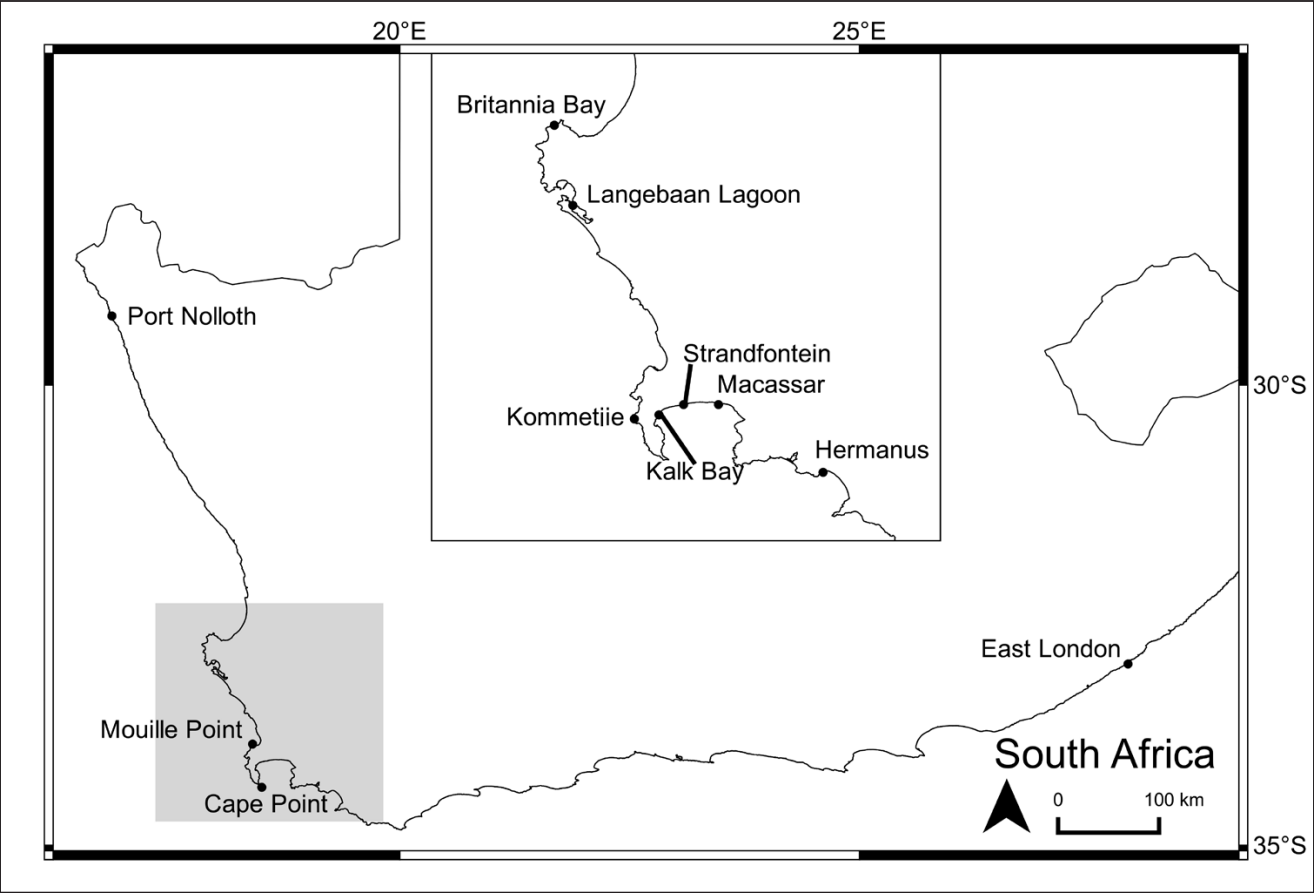
Map of South Africa demonstrating sampling localities and other locations mentioned in the text.

Where possible, 90 starfish were collected during low spring tide from intertidal rocky shores at each of four main collecting sites (Fig. 2; compiled using QGIS v.2.6.1): Kalk Bay and Hermanus (south–west coast), and Kommetjie and Britannia Bay (west coast) which lie within two bioregions. At each site, 30 specimens were collected from three vertical intertidal zones; the high–, mid– and low shores. An exception was Britannia Bay, where few starfish could be found in the lowest zone. Twelve additional specimens were added to the analysis from other sites around the coast to either enhance the *Parvulastra dyscrita* sample size, or to include *Parvulastra exigua* of unusual appearance or from unusual habitats, such as intertidal sandbanks (Table 2).

**Table 2.** Number of starfish collected per sampling location. Brackets indicate the number of *Parvulastra dyscrita* collected.

Coast	Location	High	Medium	Low	Sand	Total
West	Britannia Bay (B)	33	31	2	–	66
	Langebaan Lagoon (L)	–	–	–	9	9
	Kommetjie (Ko)	33	30	33	–	96
South–west	Kalk Bay (Ka)	30	30	34(6)	–	94
	Strandfontein (S)	–	–	2 (1)	–	2
	Macassar (M)	–	–	(1)	–	1
	Hermanus (H)	33	32	30	–	95
					Total	363

### Morphology and anatomy

The oral and aboral surface of each specimen was photographed after collection to document the color pattern of each starfish. After preservation in 70% ethanol, several qualitative and quantitative characteristics, based predominantly on previous taxonomic descriptions from both known species, were recorded, using a dissecting microscope and digital caliper respectively (Table 3). In addition, marginal plate spines and tube feet were examined, but these characteristics were excluded from further analyses, as no differences between specimens were noted. The abactinal and actinal surface coloration of each individual specimen was also eliminated while only R/r values were considered, due to the high variability observed and possibility of skewing the analyses respectively. Specimens were not retained after examination.

**Table 3.** Starfish characteristics examined per specimen and used in multivariate analyses.

Characteristic		Technique
Quantitative	R/r	Expression of body proportion. R = greater radius measured along the ambulacral groove. r = smaller, interambulacral radius. Both measured along three non–deformed arms per specimen and averaged.
	Peristomial membrane diameter	Used as an expression of body size. Measured twice per specimen and averaged.
	Madreporite diameter	Measured twice per specimen and averaged.
	Papulae diameter	Five diameters measured per specimen and averaged.
	Oral plate spines	Number of oral plate spines.
	Oral plate erect spinulation	Number of erect spines per oral plate.
	Oral marginal plate spinulation	Number of oral marginal spines.
Qualitative	Color morph	Green or mottled.
	Abactinal surface spinulation	Described as having either fine, short columnar or coarse, granuliform globose spinelets.
	Abactinal surface texture	Either clusters of spinelets, or evenly granular surface texture.
	Adradial actinal spinulation	Absent or present. Where present, noted whether these spines occurred in more, or less than, three arms.
	Furrow/Ambulacral spinulation	Classified according to relative number of plates with one spine, as well as the presence or abundance of three spines per plate.
	Actinal intermediate plate spinulation	Classified according to the relative number of plates with one or two spines, starting position of the plates with two spines and the presence of plates with three spines.
	External visible gonopore position	Having either oral gonopores or none (aboral gonopores are difficult to observe).
	Gonopore position by dissection	Dissected to determine gonad placement and definitively document gonopore position.

### Statistical analyses

**Figure 3.**
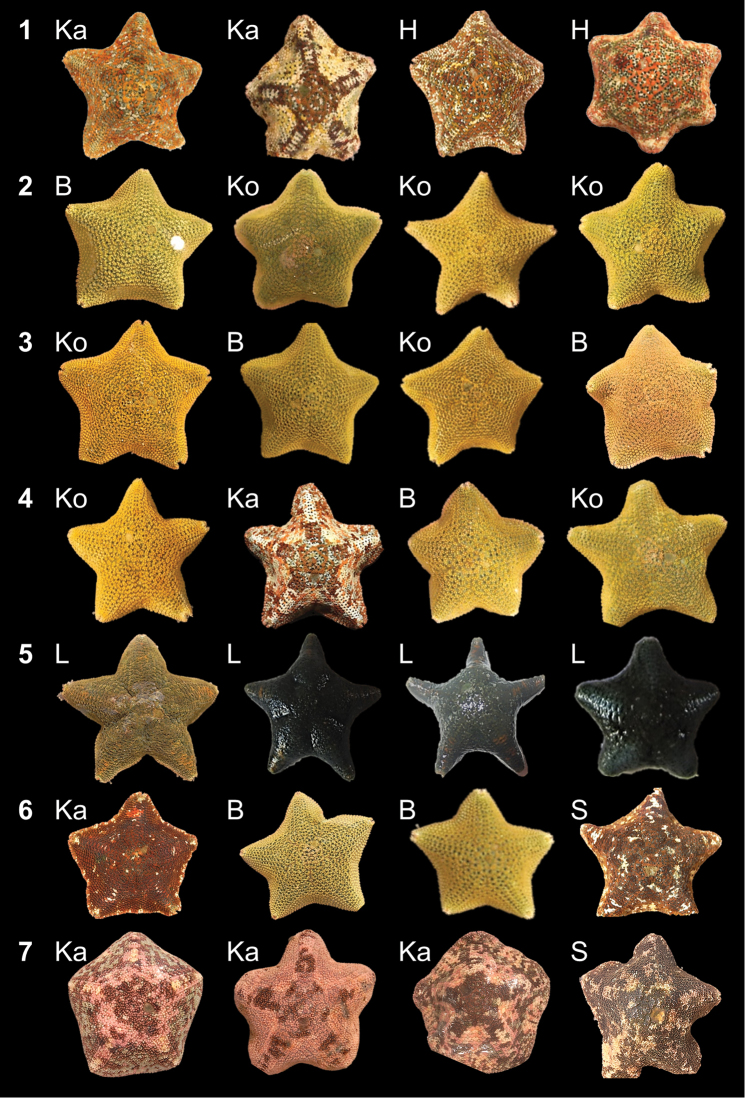
Shape and color pattern of four specimens from each of the seven groups of seven specimens as defined in Table 4, and used in the genetic analyses. Letters indicate location of specimen collection as seen in Table 2. Photos by R.P. Payne; individuals not to scale, with approximate sizes given in Table 5.

Overall, 354 specimens were included in the multivariate analyses, which were performed on unstandardised and untransformed characteristic data using PRIMER v.6.1.5 (Plymouth Routines in Multivariate Ecological Research; Clarke and Gorley 2006). A non–metric multidimensional scaling (MDS) ordination, based on a resemblance matrix generated from Bray–Curtis similarities, was used to visually assess specimen similarity. Additionally, this ordination was utilized to identify outlying individuals which were included in the genetic analyses. Six groups were defined (Fig. 3; Table 4), each comprising seven individuals that could possibly represent cryptic species within *Parvulastra exigua*. A seventh group, consisting of seven *Parvulastra dyscrita*, was included for comparison.

**Table 4.** Specimen groups selected for genetic analyses. Each group comprises seven individuals identified on an MDS.

**Group**	**Description**
1. Mottled	Individuals from south–west coast with variegated coloration.
2. Green	Individuals from west coast with green coloration.
3. Orange	Individuals from west coast with orange coloration.
4. Two oral plate spines	Individuals with two oral plate spines, as opposed to the four observed in most specimens collected.
5. Langebaan	Individuals from a sandflat habitat with deep aboral ‘dents’ and peculiar abactinal surface spinulation. Some also appear non–pentagonal.
6. Peculiar	Individuals with atypical coloration, shape, size etc.
7. *Parvulastra dyscrita*	Outgroup included for comparison.

The one–way ANOSIM (analysis of similarity) routine was performed to determine whether possible specimen groupings are associated with any of the documented characteristics, with the significance of the statistical tests assigned at the 5% level. Thereafter, SIMPER (similarity percentage analysis) was used to determine the characteristics that contribute to at least 90% of the difference between divergent cluster groups.

## Genetics

### DNA extraction, PCR and sequencing

Overall, 49 specimens were selected for genetic analyses (Fig. 3; Table 4); 44 were successfully sequenced. Approximately 25 mg of the specimen arm was removed for analysis. DNA was extracted using the NucleoSpin Tissue Kit (Machery–Nagel), following the manufacturer’s instructions.

A partial section of the mtDNA cytochrome oxidase I gene was amplified by PCR, using a combination of primers; the invertebrate primers LC01490 and HCO2198 (Folmer et al. 1994), as well as *Parvulastra exigua* specific primers: Pexig_F1 (5’–CTTTCCCACGAATGAACAAYATGAGC–3’) and Pexig_R1 (5’–CCGAGGGCTCATAGGAGAGGAGTGTC–3’) (Mertens 2012). All amplifications were performed in 25 µL reactions, with the PCR protocol as follows: an initial denaturing step of 3 min at 94 °C, followed by 35–38 cycles of 94 °C for 30 s, an annealing temperature of 45 °C for 45 s, and 45 s at 72 °C, with a final extension of 10 min at 72 °C. The number of cycles (35–38) was dependent on the primers and DNA dilution used, which differed according to specimen. PCR products were visualized on a 1% agarose gel stained with ethidium bromide, and sequences were generated on an ABI–3100 automated sequencer, at the Stellenbosch University Central Analytical Facility.

### Sequence analyses

Geneious v.6.1.6 was used to build an unrooted neighbor–joining tree with bootstrap support from consensus sequences that had a final length of 345 bp. Sequences were also analyzed using TCS v.1.21. (Clement et al. 2000) in order to generate a parsimony haplotype network. For the latter analysis, a 95% plausible connection limit was used. The parsimony haplotype network was visualized using Haploview (Barrett et al. 2005).

## Results

Of the 354 specimens included in the analysis, eight were identified as *Parvulastra dyscrita* and the remainder as *Parvulastra exigua*. The two species are morphologically distinct (Fig. 1); Table 5 lists the main morphological differences between them. The collection location, as well as several characteristics including size, abactinal surface coloration, surface spinulation, surface texture and the presence or absence of adradial actinal spines can aid in the identification of specimens in the field.

**Table 5.** Characteristics that distinguish *Parvulastra exigua* from *Parvulastra dyscrita*, based on published literature and measurements taken during the present study.

Characteristic		*Parvulastra exigua*	*Parvulastra dyscrita*	Source
Quantitative	Size; R/r	Small, up to 20 mm; 1.07–1.83	Larger, up to 40 mm; 1.16–1.45	Branch et al. (2010)
	Peristomial membrane diameter (mm)	1.26–6.10	4.70–7.15	–
	Madreporite diameter (mm)	0.19–2.92	2.27–4.62	–
	Papulae diameter (mm)	Large; 0.07–0.28	Small, numerous; 0.13–0.26	Clark (1923); O’ Loughlin and Waters (2004)
	Oral plate spines	Two, four or variable.	Four or more, often variable.	–
	Oral plate erect spinulation	Two tall oral spines per plate, often consisting of two spines in the place of one.	Two tall oral spines per plate, often consisting of ‘bunches’ of spines in the place of one.	Clark (1923); O’ Loughlin and Waters (2004)
	Oral marginal plate spinulation	Three–five spines per plate, or a combination.	Five–seven spines per plate, or a combination.	Clark (1923); O’ Loughlin and Waters (2004)
Qualitative	Abactinal surface coloration	Dull khaki–green, orange, blue, brown and orange–shouldered on the west coast of South Africa. Variegated (often geometrical) patterns on the south and east coast, including most color combinations.	Mottled shades of pale pink, white, purple and maroon.	Clark (1923); Clark and Courtman–Stock (1976); Branch et al. (2010)
	Actinal surface coloration	Variable; not consistently blue–green.	Not consistently blue–green; bluish yellow.	Dartnall (1971); Clark (1974)
	Abactinal surface spinulation	Fine, short columnar.	Coarse, granuliform globose.	Clark (1923); O’ Loughlin and Waters (2004); Branch et al. (2010)
	Abactinal surface texture	Clusters of spines.	Evenly granular.	Branch et al. (2010)
	Adradial actinal spinulation	Absent.	Often present.	O’ Loughlin and Waters (2004)
	Furrow/ Ambulacral spinulation	Two (often three) slender, short spines.	Two (often three) slender, short spines.	Clark 1923; Dartnall (1971); O’ Loughlin and Waters (2004)
	Actinal intermediate plate spinulation	Each plate with only one or two spines, with the latter occurring more frequently distally.	Many plates with two spines each, some with three.	Clark (1923); Dartnall (1971); O’ Loughlin and Waters (2004)
	Subambulacral spines	Tall, thick, pointed spine on each adambulacral plate.	Large, blunt, truncate spine on each adambulacral plate.	Clark (1923); Dartnall (1971); O’ Loughlin and Waters (2004)
	External visible gonopore position	Oral or none. Often difficult to detect.	None. Difficult to detect.	–
	Gonopore position	Oral–two in each interradius, separated from oral plates by approximately three actinal plates.	Aboral.	Dartnall (1971); Clark and Courtman–Stock (1976) O’ Loughlin and Waters (2004)

All collected *Parvulastra dyscrita* specimens had muted shades of pink, white, brown and turquoise on the abactinal surface, with a bluish–yellow color orally (Fig. 1). The gonopore position was not externally visible in any of these specimens, but once dissected it was clear that all had aboral gonopores

Representative individuals of *Parvulastra exigua* collected from the various study sites are depicted in Fig. 3, displaying a wide range of both shape and color. Specimens collected from Kommetjie and Britannia Bay (west coast sampling sites) exhibited a variety of uniform abactinal surface coloration, ranging from pale green, olive green, to orange, brown and blue, while those from Hermanus and Kalk Bay (south–west coast sampling sites) were all mottled morphs, of darker coloration. No green morphs were sampled from the south–west coast collection sites and neither were intermediate forms. Individuals collected from Langebaan Lagoon were dark green, with some exhibiting an orange shoulder. Actinal coloration was highly variable between and among sampling sites and the oral gonopore position could be seen in some preserved specimens, while no gonopores could be seen in others. Specimens collected from Langebaan Lagoon all demonstrated large aboral ‘grooves’ that could be confused for gonopores. However, when dissected, all *Parvulastra exigua* specimens displayed oral gonopores.

### Inter–species differences

**Figure 4.**
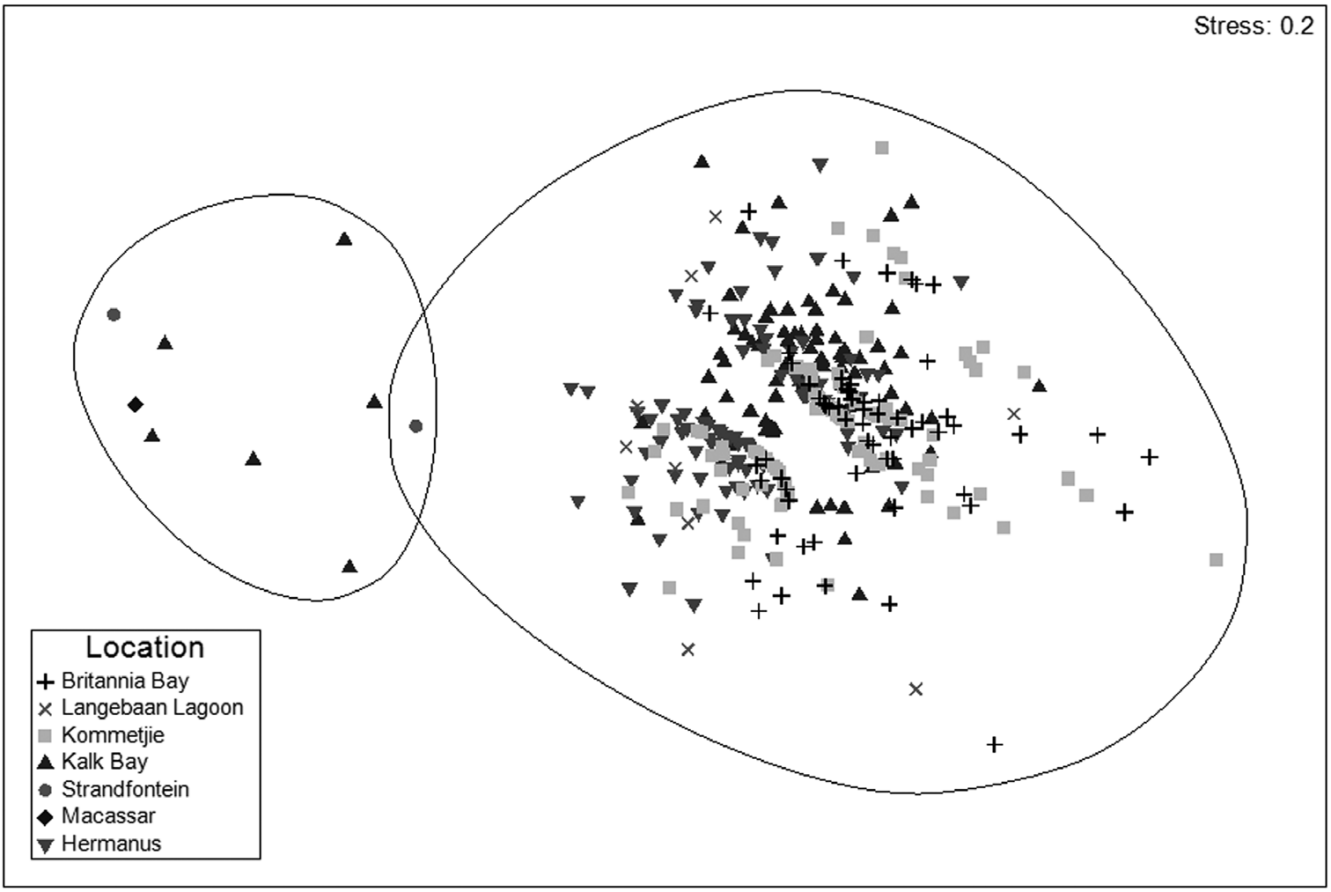
Non–metric MDS ordination of all specimens analyzed. Two clear clusters displayed which represent the species *Parvulastra dyscrita* on the left, and the species *Parvulastra exigua* on the right. Circles indicate 75% similarity. The specimen causing a cluster overlap is of the species *Parvulastra exigua*, but has many of the morphological characteristics associated with *Parvulastra dyscrita* due to its large size and possibly collection location.

**Figure 5.**
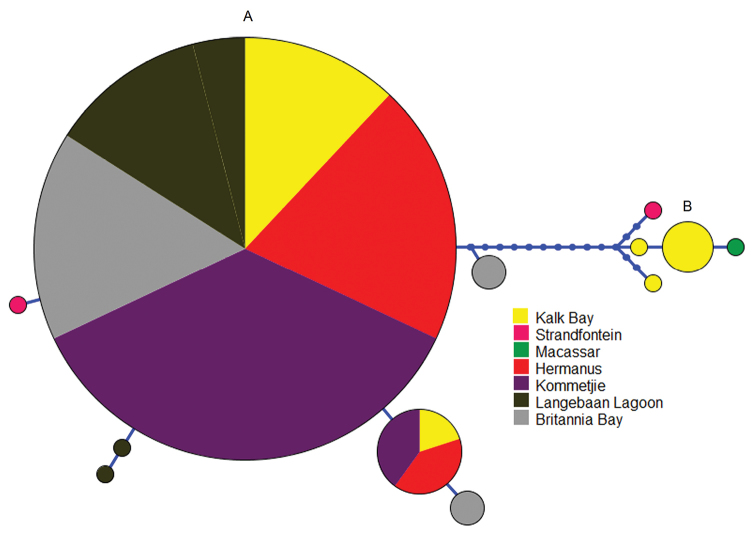
Parsimony haplotype network for (**A**) 37 *Parvulastra exigua* and (**B**) 7 *Parvulastra dyscrita* specimens. Circle size relates to the frequency of each haplotype, with color indicating origin of the individuals. Smallest circles represent one individual and one haplotype. Extinct or not sampled haplotypes are marked by a blue dot and each line represents one mutational step.

As can be identified in Fig. 4, two clear clusters, with an average dissimilarity of 35.96%, are evident in the MDS plot, representing *Parvulastra dyscrita* and *Parvulastra exigua*. This confirms that these represent distinct species, with gonopore position (ANOSIM, R = 0.981, p = 0.001), abactinal surface texture (ANOSIM, R = 0.981, p = 0.001), abactinal surface spinulation (ANOSIM, R = 0.757, p = 0.001) and oral plate spines (ANOSIM, R = 0.682, p = 0.001) playing a predominant role in this configuration. SIMPER results suggest that spinulation plays a major role in the delineation of these species, with erect oral plate, actinal intermediate plate, oral marginal plate and furrow/ambulacral spinulation contributing to 57.55% of the difference between the two species. An unrooted neighbor joining tree (data not shown) supports this distinction, with a clear separation of the sequences into two clades with 100% bootstrap support. This finding is supported by the haplotype network (Fig. 5), which failed to connect the two clades with more than 95% probability.

### Intra–species variation

Within the *Parvulastra exigua* cluster (Fig. 4), the specimens differed significantly, with oral plate spines (two to four spines per oral plate or variable, ANOSIM, R = 0.677, p = 0.001) and abactinal surface spinulation (short columnar spines to similar spines but with slightly different shape, ANOSIM, R = 0.527, p = 0.001) playing a predominant role in this configuration. Overall there seems to be little morphological separation between *Parvulastra exigua* specimens. The phylogenetic tree (data not shown) and the haplotype network (Fig. 5A) show little genetic variation between *Parvulastra exigua* sampled at different locations, tidal heights or color morph. Two main haplotypes dominate the haplotype network for *Parvulastra exigua* (Fig. 5A), both of which are well distributed across the sampling locations, with three unique haplotypes found along the west coast. The connections between the haplotypes indicate that they are genetically very similar (at the maximum five mutational steps distance). Overall, this evidence suggests that all *Parvulastra exigua* specimens collected in this study represent a single, morphologically variable species.

## Discussion

### The validity and identification of *Parvulastra dyscrita*

*Parvulastra dyscrita* and *Parvulastra exigua* show a clear separation, based on morphology, anatomy and genetics, unambiguously confirming them to be two distinct species. The differences in characteristics of the two species supports those defined in earlier taxonomic work (Table 5) with gonopore position, abactinal surface texture and abactinal surface spinulation playing a major role. SIMPER results confirm that spinulation is a main delineator between these species.

The clear separation of these two species confirms Dunbar’s (2006) results, but that study also highlights the ease with which specimens can be misidentified. This is understandable, especially in the case of large *Parvulastra exigua*, that can sometimes look morphologically very similar to *Parvulastra dyscrita* (personal observation), an example being the specimen in Fig. 4 that causes the 75% similarity circles to overlap. Based on the position of the gonopore, and several other characteristics, this specimen is definitely *Parvulastra exigua*, but is one of the largest specimens collected overall, had spinulation characteristics similar to those of *Parvulastra dyscrita* and was collected from the front of Strandfontein rockpool, which could be considered a subtidal location. All these characteristics facilitate misidentication in the field and caution that more detailed examination of spinulation and gonopore position are needed to confirm identification of ambiguous specimens.

### Species resolution in *Parvulastra exigua*

All *Parvulastra exigua* specimens collected exhibited oral gonopores, with only slight morphological differences in oral plate spines and abactinal surface spinulation, but no separation great enough to indicate the presence of a cryptic species. This was confirmed by molecular analyses; the haplotype network shows little genetic variation within *Parvulastra exigua* sampled at different localities, tidal height or specimen group. Mertens (2012) conducted genetic analyses on a further 177 *Parvulastra exigua* specimens collected ~800 km of the west coast of South Africa (from Kommetjie to Port Nolloth) and did not find evidence of any cryptic species. However, it is important to note that no intermediate reddish–orange color morphs were collected in the present study and only a few Kommetjie specimens were analyzed genetically. All of this evidence suggests that if an undescribed *Parvulastra* species exists, it occurs in very low numbers, or in a very narrow geographic range, as suggested by Dunbar (2006). This warrants further investigation, and perhaps an extensive future sampling survey at Kommetjie. In addition, the slight intraspecific variation and observed colour polymorphism in *Parvulastra exigua* may be maintained by temperature and/or predation, as suggested by Dunbar (2006), and warrants more study. Future work should also include further comparisons (both morphological and molecular) of *Parvulastra exigua* and *Parvulastra dyscrita* specimens across the entirety of their currently known distributions, as well as the potential hybridization between South African *Parvulastra* species, which has not been investigated.

### Gonopore position

After no *Parvulastra exigua* specimens from our original samples were found to have aboral gonopores, a further 200 *Parvulastra exigua* individuals were collected from Mouille Point, a location where a museum specimen with supposed aboral gonopores had been collected previously and examined by Hart et al. (2006). These specimens were also found to all have oral gonopores. In addition, Dunbar (2006) also found an examined subset of her *Parvulastra exigua* specimens to only have visible oral gonopores. On enquiry, it was determined that Hart et al. (2006) only examined museum specimens externally (Michael Hart and Maria Byrne, pers. comm.) and it is suggested that they mistook abactinal dimples (or the lack of visible oral gonopores) for aboral gonopores. This proposal is supported by the fact that *Parvulastra dyscrita*, a species with aboral gonopores, has no easily identifiable external gonopore position, making it hard to confirm gonopore position without dissection. A similar situation was faced with regards to the few *Parvulastra exigua* specimens examined in this study, as these displayed no oral gonopores and had to be dissected in order to reveal that the gonads were indeed orally directed. Finally, Langebaan Lagoon *Parvulastra exigua* specimens have deep abactinal grooves, and as some lack visible oral gonopores unless dissected, they could easily be mistaken as having aboral gonopores. It is also important to note that Waters and Roy (2004) did not include *Parvulastra dyscrita* in their molecular analysis and so some of the samples included in their analysis that depicts cryptic diversity in South Africa, could include *Parvulastra dyscrita* misidentified as *Parvulastra exigua* (Michael Hart, pers. comm.). Thus, this study suggests that *Parvulastra exigua* specimens in South Africa with aboral gonopores do not exist.
